# Oxidative Dysregulation in Early Life Stress and Posttraumatic Stress Disorder: A Comprehensive Review

**DOI:** 10.3390/brainsci11060723

**Published:** 2021-05-29

**Authors:** Evangelos Karanikas, Nikolaos P. Daskalakis, Agorastos Agorastos

**Affiliations:** 1 Department of Psychiatry, 424 General Military Hospital, GR-56429 Thessaloniki, Greece; epkarani@yahoo.com; 2 II. Department of Psychiatry, Division of Neurosciences, School of Medicine, Aristotle University of Thessaloniki, GR-56430 Thessaloniki, Greece; 3 Department of Psychiatry, McLean Hospital, Harvard Medical School, Belmont, MA 02478, USA; ndaskalakis@mclean.harvard.edu; 4 VA Center of Excellence for Stress and Mental Health, VA San Diego Healthcare System, La Jolla, San Diego, CA 92161, USA

**Keywords:** posttraumatic stress disorder (PTSD), stress, trauma, oxidative stress, antioxidants, inflammation, oxidation, redox state, redox system, early life stress, childhood adversity, immune system, reactive oxygen species, reactive nitrogen species, oxidative phosphorylation, nitric oxide, glutamate, oxidative enzymes

## Abstract

Traumatic stress may chronically affect master homeostatic systems at the crossroads of peripheral and central susceptibility pathways and lead to the biological embedment of trauma-related allostatic trajectories through neurobiological alterations even decades later. Lately, there has been an exponential knowledge growth concerning the effect of traumatic stress on oxidative components and redox-state homeostasis. This extensive review encompasses a detailed description of the oxidative cascade components along with their physiological and pathophysiological functions and a systematic presentation of both preclinical and clinical, genetic and epigenetic human findings on trauma-related oxidative stress (OXS), followed by a substantial synthesis of the involved oxidative cascades into specific and functional, trauma-related pathways. The bulk of the evidence suggests an imbalance of pro-/anti-oxidative mechanisms under conditions of traumatic stress, respectively leading to a systemic oxidative dysregulation accompanied by toxic oxidation byproducts. Yet, there is substantial heterogeneity in findings probably relative to confounding, trauma-related parameters, as well as to the equivocal directionality of not only the involved oxidative mechanisms but other homeostatic ones. Accordingly, we also discuss the trauma-related OXS findings within the broader spectrum of systemic interactions with other major influencing systems, such as inflammation, the hypothalamic-pituitary-adrenal axis, and the circadian system. We intend to demonstrate the inherent complexity of all the systems involved, but also put forth associated caveats in the implementation and interpretation of OXS findings in trauma-related research and promote their comprehension within a broader context.

## 1. Introduction

Stress is defined as the state of threatened homeodynamic balance of the organism by intrinsic or extrinsic, real or perceived altering conditions or stimuli, known as stressors [1,2]. The organism’s total response to these stressors is defined as allostatic load and mirrors an active process in the direction of stabilization. Ephemeral and motivating stress states lead to response habituation and are rather beneficial, while inadequate, aversive, excessive (i.e., traumatic) or cumulative stress states may exceed the organism’s natural adaptive capacity and significantly affect homeostatic responses [1,3]. Traumatic stress exposure, especially in critical developmental periods of heightened stress sensitivity and plasticity (e.g., early childhood), may over- or under-sensitize neuroendocrine responses, leading to a chronically altered homeodynamic state (i.e., *cacostasis*) and a vulnerable phenotype, characterized by disrupted stress reactivity and accumulated allostatic load [4,5]. Several types of traumatic stress exposure (Table 1) may thus gradually affect master homeostatic systems at the crossroads of peripheral and central (brain) susceptibility pathways and lead to the biological embedment of the psychological stress through neurobiological alterations even decades later, with profound, cumulative, and debilitating effects for health, overall disease vulnerability, physical and mental co-morbidity, as well as all-cause mortality [3,4,5,6,7,8,9]. However, although mounting epidemiological evidence supports the causal relationship between traumatic stress and its long-term adverse health-related effects, less is known about the exact allostatic trajectories through which trauma is translated into health risk.

Stress, in general, has been suggested to etiologically relate to oxidative mechanisms [17] and the same presumption applies also for psychological stress [18,19]. Lately, there has been an exponential knowledge growth concerning the effect of traumatic stress on oxidative components and redox-state homeostasis [20,21,22]. The neurobiological substrates of the redox system become activated under conditions of severe and chronic stress with excessive production of oxidative stress (OXS) and/or inadequacy of the organism’s antioxidant capacity [23]. In this process, mitochondria become dysfunctional, and vital constituents of the cell such as protein, lipids, DNA end up degraded, finally leading the cell to an apoptotic state. This extensive review encompasses a detailed description of the oxidative cascade components along with their physiological and pathophysiological functions and a systematic presentation of genetic and epigenetic regulation of trauma-related OXS in preclinical and clinical studies, followed by a substantial synthesis of the involved oxidative cascades into specific and functional, trauma-related pathways. Particular emphasis was drawn on early life stress (ELS) (i.e., adverse childhood experiences, ACEs; childhood trauma, CT) and posttraumatic stress disorder (PTSD) and their oxidative neurobiological substrate. Finally, in our attempt to highlight discrepancies and delineate obscurities of the literature of trauma-related oxidative neurobiological trajectories, we expanded on and suggested possible oxidative regulatory mechanisms as well as implications for their interplay with other inflammatory mechanisms. The ultimate goal of the current review is to help structure novel research avenues within psychosomatic medicine by identifying complex trauma-induced oxidative mechanisms and confounding factors [10,11,12,13,14,15,16].

## 2. Oxidative Stress

### 2.1. Oxidative Phosphorylation

In eukaryote cells of aerobic organisms, the energy stored within nutrients is released through enzymatic oxidation in order to produce adenosine triphosphate (ATP). This metabolic pathway is called oxidative phosphorylation and embraces a transfer of electrons from electron donors to electron acceptors (i.e., oxygen) in redox reactions, which creates energy used to transform adenosine diphosphate (ADP) to ATP in a phosphorylation reaction [24]. Over 90% of the ATP required for the regular cell function is provided by the aerobic respiratory electron transport system of mitochondria (encompassing five multi-subunit enzyme complexes, Complex I–V) [25], while additional oxidation processes involve cytoplasmatic enzyme activity [e.g., nicotinamide adenine dinucleotide phosphate (NADPH) oxidase (NOX), xanthine oxidase, and cytochromes P450] [24,26,27]. Both systems (i.e., mitochondrial and cytoplasmatic), can result in the production of free radicals, namely reactive oxygen species (ROS) [e.g., superoxide anion (O_2_^●−^), hydrogen peroxide (H_2_O_2_), hydroxyl radical (OH)], and reactive nitrogen species (RNS) [e.g., peroxynitrite (ONOO^−^), nitric oxide (NO)] [24]. However, other processes, such as activation of the immune system, lipid peroxidation, ischemia, and trauma, can also contribute to ROS/RNS generation [28,29].

### 2.2. The Physiological Role of ROS/RNS

ROS/RNS, in addition to being byproducts of ATP-generating chemical reactions, have a vital role in the organism’s life and survival. Cell signaling, signal transduction, neuronal differentiation, mitosis, and cellular response to injury and infections are only some of the physiological functions served by ROS/RNS constituents [28]. In particular, ROS effects in the central nervous system (CNS) can include transient changes in neuronal activity and synaptic plasticity, as well as diverse actions on both excitatory and inhibitory neurotransmission, including regulatory changes on neurotransmitter receptors, long-term potentiation, learning, memory, and gene expression [30,31,32,33,34,35,36], suggesting a role in normal brain physiology. Similarly, RNS are also involved in the regulation of neuronal excitability, induce several forms of synaptic plasticity, and interfere with neurotransmission [37,38].

### 2.3. Redox Imbalance and OXS

Normally, ROS/RNS levels are counter-regulated to optimum levels by enzymatic antioxidants [e.g., superoxide dismutase (SOD), catalase (CAT), glutathione peroxidase (GSH-Px), glutathione reductase (GSH-Rd), thioredoxin (TXN), thioredoxin reductase (TXN-Rd), peroxiredoxin (PRX), paraoxonase (PON)-1] and non-enzymatic [e.g., glutathione (GSH), vitamins A, C, and E, selenium, lipoic acid, L-cysteine, L-carnitine, L-carnosine, homocysteine, retinol, a-tocopherol, heme oxygenase (HO)-1, ubiquinol, flavonoids, and carotenoid] antioxidants [24,39,40].

**Figure 1:**
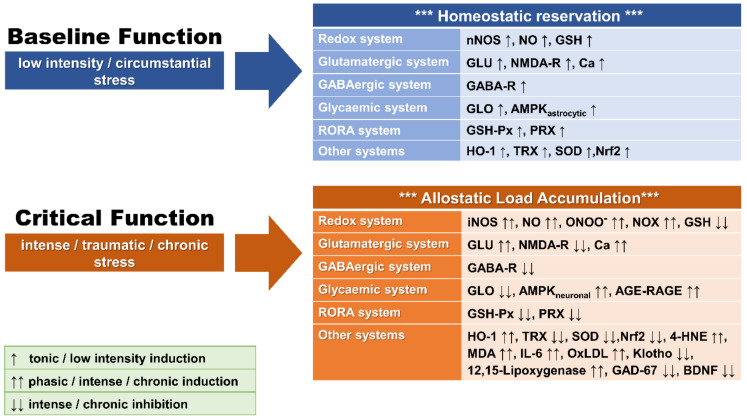
Graphical representation of baseline and stress-associated altered function of basic redox homeostatic systems leading to OXS. 4-HNE: 4-hydroxynonenal; AGE: advanced glycation end-products; AMPK: 5′-adenosine monophosphate-activated protein kinase; Ca: calcium; GABA-R: γ-amino-butyric-acid receptor; GAD-67: glutamic acid decarboxylase-67; GLO: glyoxalase; GLU: glutamate; GSH: glutathione; GSH-Px: GSH peroxidase; HO-1: heme oxygenase-1; IL-6: interleukin-6; MDA: malondialdehyde; NMDA-R: N-methyl-D-aspartate receptor; NO: nitric oxide; NOS: nitric oxide synthase; iNOS: inductible NOS isoform; nNOS: neuronal NOS isoform; Nrf2: nuclear related factor-2; ONOO^−^: peroxynitrite; OxLDL: oxidized low-density lipoprotein; PRX: peroxiredoxin; RAGE: AGE receptors; SOD: superoxide dismutase; TRX: thioredoxine reductase. ↑: low-level induction; ↑↑: high level induction: ↓↓: high-level inhibition.

**Figure 2:**
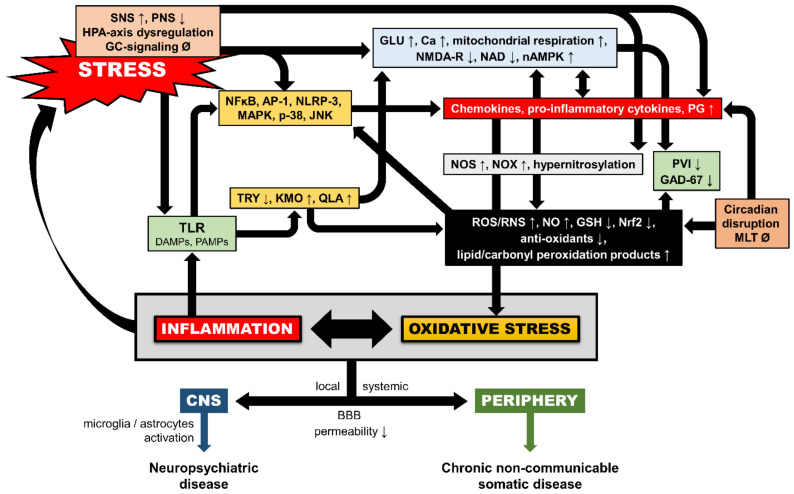
Schematic model of multilevel interactions between inflammatory and redox pathways under toxic stress exposure. AP-1: activator protein-1; BBB: blood-brain-barrier; Ca: calcium; DAMPs: damage-associated molecular patterns; GAD-67: glutamic acid decarboxylase-67; GC: glucocorticoid; GLU: glutamate; GSH: glutathione; HPA-axis: hypothalamic-adrenal-pituitary axis; JNK: c-Jun N-terminal kinase; KMO: kynurenine 3-monooxygenase; MAPK: mitogen-activated protein kinase; MLT: melatonin; NAD: nicotinamide adenine dinucleotide; NFkB: nuclear factor kappa beta; nAMPK: neuronal 5′-adenosine monophosphate-activated protein kinase; NLRP-3: nod-like receptor protein-3; NMDA-R: N-methyl-D-aspartate receptor; NO: nitric oxide; NOS: nitric oxide synthase; NOX: nicotinamide adenine dinucleotide phosphate (NADPH) oxidase; Nrf2: nuclear related factor-2; p-38: protein-38 MAPK; PAMPs: pathogen-associated molecular patterns; PG: prostaglandins; PNS: parasympathetic nervous system; PVI: GABAergic parvalbumin interneurons; QLA: quinolinic acid; RNS: reactive nitrogen species; ROS: reactive oxygen species; SNS: sympathetic nervous system; TLR: toll-like receptors; TRY: tryptophan; ↑: induction; ↓: inhibition; Ø: no change.

When ROS/RNS levels increase beyond the organism’s antioxidant system’s repairing (i.e., detoxifying) capacity, OXS arises [41]. The detrimental OXS effects can escalate to damage all molecular components of the cell, including lipids, proteins/enzymes, carbohydrates, and nucleic acids (i.e., DNA/RNA damage or strand breaks), resulting in functional cellular alterations and, finally, cell apoptosis and necrosis (Figure 1) [42,43,44]. Oxidative stress plays an essential role in the pathogenesis of numerous chronic diseases such as cardiovascular diseases, diabetes, accelerated cellular aging, neurodegenerative diseases, and cancer [45]. The brain is particularly known for a combination of high oxygen consumption, its lipid-rich constitution, the presence of redox-active metals (Cu and Fe), and modest antioxidant defenses [46,47,48]. OXS-related harmful consequences in the CNS include an increase of blood-brain-barrier (BBB) permeability, disruption of neurogenesis, impairment of synaptic plasticity, alterations of neurotransmission, remodeling of neural morphology, and cellular signaling in general [15,18,49], and have been associated with a number of neuropsychiatric disorders (Figure 2) [50,51,52].

### 2.4. Redox System Components

#### 2.4.1. Nitric Oxide (NO) and the Oxidative Phosphorylation Cascade

As the main component of the oxidative phosphorylation process, NO plays a central role in the generation of OXS [53]. In homeostatic conditions, NO in low concentrations mainly serves neurotransmission and cellular protection (through GSH increase) [54]. In case of higher energy demand and increased neuronal activity such as in stress, hypoxia, inflammation/injury, and NMDA stimulation, Ca transients together with the 5’ AMP-activated protein kinase (AMPK) cascade lead to increased glycolysis and catabolic processes (e.g., fatty acid oxidation) and induction of the inducible isoform of nitric oxide synthase (iNOS) in order to sustain neuronal activity [55]. iNOS induction leads to dysregulation of the NO equilibrium, ONOO^−^ formation, and GSH trafficking decline, collectively causing mitochondrial damage via ROS/RNS generation [56]. Apart from NO, other components of the oxidative phosphorylation cascade such as the HO-1 and GSH pathways and lipid oxidation products (e.g., 4-hydroxynonenal, 4-HNE) are also involved in the generation of ROS (i.e., through pro-oxidant Fe generation) and the promotion of autophagy, senescence, cell cycle arrest, apoptosis, and cell death [57,58,59,60].

#### 2.4.2. Glutamate

Glutamate (GLU) is related to the oxidative phosphorylation cascade via containment of modulatory redox sites [61]. Short-lasting, acute stress requires a physiological level of GLU excitatory neurotransmission for a rapid adaptive reaction [62]. However, emotional stress is associated with excessive GLU release, which combined with decreased GLU reuptake into astrocytes can lead to NMDA-R downregulation/dysfunction, NOX expression upregulation, and excitotoxicity [63]. Elevated NOX activity produces ROS, which further enhances GLU release, GSH decrease, and NMDA-R impairment (oxidation) in a feed-forward fashion [64]. In addition, excessive GLU release triggers IL-6 production, and in turn, pro-inflammatory cytokines can activate the NOX-mediated oxidative cascade [65], thus completing another parallel pathophysiological sideway.

#### 2.4.3. Intracellular Calcium (Ca)

Intracellular Ca signaling is directly related to cellular oxidative excitotoxicity, as it is majorly involved in several cellular survival/apoptotic pathways [e.g., GSH, TRX, PRX, cyclic-AMP response element binding protein (CREB), and forkhead box O (FOXO) pathways] [66,67,68,69] and an important regulator of transfer molecules (e.g., hydrogen peroxide) at endoplasmic reticulum-mitochondria contact sites [70]. An intense intracellular Ca “overload”, consequent to increased energy demand, may trigger NOS activation, leading subsequently to excessive formation of NO and finally to OXS [71], and affects NMDA-R function possibly via the decline of Ca/calmodulin-dependent protein kinase II activity [72,73].

#### 2.4.4. N-Acetylaspartate

N-Acetylaspartate (NAA) is a free amino-acid produced by mitochondria and is used as a marker for neuronal health [74,75] and a proxy for overall neuronal density [76]. NAA plays a protective regulatory role, especially in brain areas involved in traumatic stress neurobiology (e.g., hippocampus) [77], while it serves as a reservoir for GLU and its depleted state has been associated with GLU excitotoxicity [78,79].

#### 2.4.5. GABA

γ-Amino-butyric-acid (GABA) is a major inhibitory neurotransmitter in multiple levels of the CNS [80]. In particular, GABAergic parvalbumin interneurons (PVI) show high energy demand to support high-frequency neuronal synchronization, thus requiring optimal mitochondrial function [81,82] accompanied by enhanced oxidative phosphorylation [83]. In addition to the PVI, GABA_A_-R are involved in the redox equilibrium. For example, GSH potentiates GABA_A_-R function [84]. Similarly, both ROS/RNS and antioxidants (e.g., vitamin C) also exert dynamic modulation of the GABA_A_-R function [82]. Thus, ROS, in addition to extracellular GLU increase, GSH decrease and NMDA-R impairment, may lead to a dramatic decrease of both the inhibitory PVI and Glutamic Acid Decarboxylase (GAD)-67, the major GABA-synthesizing enzyme in the cortex [64].

#### 2.4.6. The RORA System

The retinoid-related orphan receptor alpha (RORA) system is thought to act as a constitutive activator of transcription by binding to the ROR response element of target genes and is particularly expressed in the prefrontal cortex, hippocampus, and hypothalamus of the CNS [85,86]. RORA is believed to be activated during OXS, thus protecting neurons through the increase of the GSH-Px1 and PRX genes expression [87].

#### 2.4.7. 12/15-Lipoxygenase

The 12/15-Lipoxygenase enzyme (transcribed by the ALOX-12 and ALOX-15 genes) participates in oxidative neurodegenerative mechanisms by activating ROS generation and supporting an oxidative attack against mitochondria in GSH-depletion conditions [88].

#### 2.4.8. GLO System

The glyoxalase (GLO) system is comprised by the glyoxalase-I and glyoxalase-II enzymes, which are responsible for the detoxification of the highly reactive dicarbonyl compound glycolytic byproduct methylglyoxal (MGO) [89,90]. MGO along with other advanced glycation endproducts AGEs, such as 3-Deoxyglycosone and Glyoxal (collectively termed as a-oxoaldehydes), can be formed from different stages of the glycation process [91]. AGEs interaction with their receptors (RAGE) induces signal transduction pathways through oxidative process culminating in NOX-dependent OxS with detrimental effects on GSH content, SOD activity (decrease), and MDA concentration (increase) [92]. GLO, thus, has a role in regenerating GSH, and its downregulation imposes detrimental effects on oxidative balance [93,94].

#### 2.4.9. Klotho Gene

Klotho is an aging suppressor gene, whose protective variant inhibits lipid oxidation, causes DNA damage (lower urinary 8-OH-DG), and strengthens the cellular antioxidant capacity through up-regulation of the Manganese SOD or SOD-2 genes expression, consequently increasing the organism’s resilience to trauma-related oxidative aberrations [95,96]. The defective SNP variant, on the other side, exhibits close associations with psychopathology and mental diseases [97].

### 2.5. Measurement of OXS

The direct measurement of free radicals is difficult due to their short half-lives and low concentrations. Thus, the quantification of OXS takes place through measurements of the ROS/RNS metabolites’ levels, antioxidants levels, as well as antioxidant enzymes’ activities and related gene expression [98]. Alternative ways to index the total antioxidants potential is by measuring the total antioxidant capacity (TAC); the total-radical non-enzymatic antioxidant potential (TRAP, representing the collective non-enzymatic antioxidant capacity of the active free radical scavengers such as GSH, Bilirubin, Vitamin E, Vitamin C, and Uric Acid); the oxidative stress index (OSI); and the total oxidant status (TOS) [98]. Another indirect way of measuring OXS utilizes the evaluation of markers of oxidative damage by assessing lipid peroxidation, protein, and DNA oxidation products. In particular, lipid peroxidation is assessed through HNE, malondialdehyde (MDA), isoprostanes, thromboxane B2 (TXB2), and thiobarbituric reactive substances (TBARS) levels [99]. Protein and DNA oxidation are assessed through protein carbonyls, 8-hydroxydeoxyguanosine (8-OH-DG), and 8-hydroxyguanosine (8-OH-G) levels, respectively [99]. Finally, both measurement of telomere length and DNA methylation age constitute important indirect indexes of OXS and cellular senescence, based on the knowledge that the latter can accelerate epigenetic aging [100,101,102].

## 3. OXS and Traumatic Stress

The bulk of the evidence suggests a systemic pro-/anti-oxidative mechanism dysregulation following traumatic stress exposure, respectively leading to systemic OXS accompanied by toxic oxidation byproducts [20,21,22,23]. Yet, there is substantial heterogeneity in research findings, probably relative to several confounding parameters (i.e., age of traumatization, time since trauma, other trauma-related parameters, etc.), as well as to the equivocal directionality of the involved oxidative and other homeostatic pathways and the specific OXS measures assessed.

### 3.1. Animal Studies

Animal models provide useful evidence for oxidative mechanisms concerning conditions mimicking ELS, SLS, and traumatic stress [i.e., maternal separation (MS), prolonged predator exposure (PPE), predator-scent stress (PSS), intense fear, single prolonged stress (SPS), repeated stress exposure (RSE) inescapable foot shocks (IFS), social isolation (SI), maternal care deprivation (MCD), prenatal maternal stress (PMS), and other stress models].

#### 3.1.1. OXS in Animal Models of ELS

Numerous animal studies using ELS models offer results suggesting increased OXS markers and reduced antioxidant activity. For example, a study on the impact of MS across three stages of the lifespan (adolescent, adult, and aged) on the mitochondrial activity in peripheral blood mononuclear cells (PBMCs) in rats showed disrupted cell cycle and long-term increases in mitochondrial activity, as well as increased sensitivity to H_2_O_2_-induced oxidative stress in vitro in adolescent and adult rats [103]. Another study on the effects of MS on the oxidative status in adult mice, showed increased plasma TBARS and decreased catalase activity in the hippocampus [104]. MS in male rats and mice has been also shown to be associated with higher ROS and mitochondrial glutathione levels in the cardiac tissue of the animals [105], as well as with significant endothelial dysfunction and increased superoxide production through higher expression of the NADPH oxidase subunits, NOX2 and NOX4, in adult animals [106]. Similarly, MS studies on OXS in reproductive tissues of the adult animals have shown epithelial alterations in combination with increased ROS production; decreased concentrations of GPx and ATP; and increased apoptosis associated with decreased count, morphology, and viability of spermatozoa in male [107]; lower ovarian tissue survival, antrum formation, ovulation, and oocyte maturation; and lower total antioxidant capacity level as defined by superoxide dismutase, GSH-Px and CAT in female mice [108].

A number of preclinical studies suggested a causative association between GABAergic PVI system disturbances with ELS, such as PMS [109], MS [110], and SI [111]. Another ELS study using an animal model of early SI in rats, revealed decreased activity of antioxidant enzymes CAT, GSH-Px, SOD, and the total antioxidant capacity, but increased levels of hydrogen peroxide in certain brain regions, of which prefrontal cortex and hippocampus were most vulnerable [112]. In another animal model, ELS induced by MCD was associated with increased protein carbonyl levels and decreased SOD and CAT activity in the brain in the early postnatal phase of the animals [113], while other studies also showed similar long-term alterations in the basal antioxidant defenses following ELS [114].

#### 3.1.2. OXS in Animal Models of PTSD

Similar results of increased OXS markers and reduced antioxidant activity can be found in a large number of animal studies using PTSD models. For example, PTSD animal models of PPE in rats showed increased lipid peroxide levels and a higher number of neuronal NOS (nNOS) positive neurons in the amygdala [115,116], as well as a higher number of nNOS positive neurons and increased NOX levels in the PFC rat brains after exposure [116]. Similar studies suggest a dose-response relationship between the duration of exposure and/or the animal freezing time during PPE and levels of ROS and other by-products of OXS in the post-mortem analysis of hippocampal and PFC rat brain tissues [117].

Additional animal studies utilizing a SPS model of PTSD in rats, also reported (i) decreased SOD activity; (ii) increased expression of NOX-2 subunits; (iii) increased MDA and phosphorylated AMPK; (iv) decreased GSH levels in amygdala and hippocampi; (v) elevated IL-6 and IL-1beta levels; (vi) higher expression of iNOS and p-p38 in hippocampi; (vii) higher cyclooxygenase-2 (COX-2) mRNA and protein expression, TNF-α, IL-6, prostaglandin E2 (PGE2), and NO levels; and (viii) cell apoptosis in the hippocampi of stress exposed animals [118,119,120,121]. In an IFS rat model of PTSD, the stress-exposed group showed a significant up-regulation of NOX2 and 8-OH-DG levels and a down-regulation of glutamic acid GAD-67 and parvalbumin in the animals’ hippocampi [122].

In RSE PTSD models in rats, RSE was associated with significantly increased hippocampal NOX and iNOS levels after exposure [123], followed by a reactive downregulation of hippocampal NMDA receptors and dysregulation of inhibitory GABA pathways. The role of NO in neuronal toxicity and its regulation by GLU and GABA thus have important implications in stress-related hippocampal degeneration [124].

Further animal models of PTSD, using combined predator and social defeat paradigms in rats, verified prior results, showing that stress exposure was markedly associated with ROS levels in PFC and hippocampi [125], and additionally reported body weight gain, increased body temperature, as well as inflammatory and fibrotic histopathologies and transcriptomic changes of heart and liver tissue, elevated inflammation and OXS-related proteins in plasma that endure over time [126]. Finally, SOD-2 gene emerged as a top gene distinguishing vulnerable from resilient animals in a PSS PTSD model [127].

#### 3.1.3. Summary and Considerations on Preclinical Animal Studies

The majority of the preclinical studies using PTSD animal models show increased stress-related OXS parameters or reduced antioxidant function associated with neuronal toxicity and degenerative pathophysiology in brain areas vital for PTSD development, such as amygdala, PFC, and hippocampus [128]. Taken together, the majority of preclinical studies suggest that stress-related conditions tend to cause depletion of the antioxidants’ levels/function as well as an increase in the OXS byproducts concentrations (Table 2). However, caution in the translation of oxidative mechanisms in humans is crucial since different stress paradigms may elicit different results. Indeed, the nature; intensity; duration of the imposed stressor; as well as the choice for measurement of the particular oxidative vs. antioxidant substance (i.e., its location within the oxidative/antioxidant cascade), the antioxidants’ levels vs. activities, and their equivocal topography in the brain can account for heterogeneity in results. For example, two animal studies using the MS [129] and SI paradigm [130] reported elevation of the antioxidants’ (CAT, GSH-Px, SOD) activity. The field is further obscured by the findings of another preclinical study on social isolation, suggesting decreased activity of GSH-Px and compromised GSH-Rd in rats’ hippocampi [131].

### 3.2. Human Studies

#### 3.2.1. OXS in Human ELS Studies

A basic difficulty characterizing the human studies on ELS-related OXS is the fact that the majority of them rely on the retrospective recall of ELS characteristics by the participants. Despite this bias, the majority of the evidence suggests a significant association of ELS with activation of pathophysiological oxidative pathways.

For example, the history of CT in healthy adolescents has been found to be related to increased OXS protein byproducts (carbonyl levels) and elevated SOD levels, as well as decreased TRAP and GSH-Px levels in plasma of healthy adolescents with CT history [132]. Similarly, plasma levels of 8-OH-DG, a DNA byproduct and OXS biomarker, were found increased in a group of patients diagnosed with personality disorder with a history of CT compared with Healthy Controls (HC) [133]. In the same study, 8-OH-DG was correlated significantly with C-Reactive Protein (CRP), which in turn was correlated with the levels of CT. In another study of healthy female adolescents recruited through youth services, the history of four or more ACEs was significantly related to elevated F2t-isoprostanes (IsoPs) levels [134]. Following the same line of evidence, a recent study conducted on early psychotic patients showed that in patients with history of CT, high peripheral GSH-Px activity was associated with smaller hippocampal volumes and higher levels of positive and disorganized symptoms [135].

The impact of severe stress in childhood on oxidative status in adulthood and biological aging has also been supported by studies assessing telomere length and mitochondrial DNA (mtDNA) copy number. Numerous studies support the fact that ELS and CT are independently associated with both shorter telomere length or increased mtDNA copy number in adults [102,136,137]. Interestingly, such studies have allowed a better differentiation of the biological pathways leading to OXS in adulthood and offered evidence that type and intensity of CT [138], age of exposure [139], memory of the trauma [140], and gender [141] all play a very important modulating role, while, for example, intensive parenting may play a biologically buffering role on telomere erosion effects of CT/ELS [142].

#### 3.2.2. OXS in Human PTSD Studies

A large portion of the literature on trauma-related oxidative mechanisms in humans comes from studies on combat-exposed veterans. For example, American Gulf War veterans showed increased cerebrospinal fluid (CSF) levels of RNS NO/ONOO^–^ [143], while Croatian war veterans with PTSD, demonstrated decreased levels of SOD and GSH-Px activities in erythrocytes compared to counterparts without PTSD [144], although already being on medication. In another metabolomics study including two cohorts of veterans with PTSD and control subjects, results showed a significant difference of certain glycerophospholipids between the two groups, which are involved in inflammation, mitochondrial dysfunction, membrane breakdown, oxidative stress, and neurotoxicity [145]. Previously, exposure to a disastrous earthquake has been associated with an increase in lipid peroxidation byproduct MDA level in serum as well as a decrease in antioxidant PON-1 activity compared to non-exposed controls [146]. In the same study, exposed individuals with an earthquake-related PTSD diagnosis showed higher MDA serum level and decreased PON-1 activity in comparison to exposed, non-PTSD survivors. Similarly, another study, although not finding any difference in the antioxidants’ activities between the PTSD subjects and HC [147], reported a positive association between the activities of antioxidant enzymes (GSH-Px and SOD) and PTSD symptoms. Finally, GLU dysregulation in specific brain areas involved in PTSD development (e.g., amygdala, PFC, dorsal anterior cingulate, hippocampus) has been repeatedly implicated in prefrontal connectivity alterations and cognitive-affective dysregulation seen in PTSD [79,148,149,150].

#### 3.2.3. OXS-Related Genetic Findings in PTSD

In addition to the above-mentioned findings, (epi)genetic studies also offer additional, yet indirect, evidence of the close relation between traumatic stress and OXS mechanisms. For example, the ALOX-12 gene (involved in the generation of 12/15-Lipoxygenase) has been suggested to be involved in veterans’ vulnerability to the PTSD, as a novel ALOX-12 locus (rs1042357/rs10852889) has been found to moderate the association between PTSD and reduced thickness of the right prefrontal cortex [151]. Another study on the Glutathione S-Transferase Mu (GSTM)-1 gene suggested its potential to predict the subsequent appearance of PTSD symptoms before deployment in a US Marines cohort, [152]. Later on, in a follow-up study based on the same cohort, Tylee and colleagues [153] showed that the PTSD diagnostic status could be predicted with 80% accuracy using an algorithm based entirely on the expression of GSTM-1 and its counterpart GSTM-2. In a study of a cohort of Vietnam veterans and matched controls investigating two genes of the NO pathway (NOS1AP and NOS1) for a potential involvement in PTSD, polymorphisms of both genes were associated with PTSD severity, stress, and resilience, supporting the key role of the NO pathway in PTSD pathophysiology and its comorbidities [154].

Furthermore, the results from the first genome-wide association study (GWAS) on PTSD suggested a single significant association between the single nucleotide polymorphism (SNP) rs8042149 in the RORA gene and the diagnosis of PTSD among veterans [155]. Later on, the results of this study were extended by Lowe and colleagues [156], demonstrating a correlation between another RORA SNP rs893290 and PTSD symptom trajectories over time in subjects with CT history.

Finally, many PTSD studies also report on the effects of PTSD on telomere length. Indeed, a recent meta-analytic review of the evidence including 3851 participants suggested a robust association between PTSD and shorter telomere length [157]. For example, in a recent study of Kang and colleagues on combat-exposed veterans, the interaction of trauma severity and PTSD status predicted for telomere length, with subjects with PTSD showing shorter telomere length and larger amygdala, than non-PTSD veterans [158]. Similarly, another recent study reported not only shorter relative leukocyte telomere length in patients with PTSD, but also higher frequency and the carriage rate of the telomerase TERT gene allele [159]. Last but not least, a gene expression study on survivors from an air show catastrophe reported evidence of down-regulated antioxidant SOD-1 and TXN-1 gene activity in patients with PTSD [160].

#### 3.2.4. Summary and Considerations in Human ELS and PTSD Studies

The majority of the human studies assessing the effect of ELS and PTSD on the oxidative equilibrium showed associations between ELS and PTSD with increased oxidation activity later in life, increased OXS byproducts generation, and/or reduced antioxidant function in the periphery, but also in brain areas vital for PTSD development (Table 3). However, several confounding factors merit discussion. For example, a study on Korean male veterans of the Vietnam war suggested that the magnitude of traumatic stress exposure also plays a vital role in this association, as findings suggested that there is an association between PTSD and shorter telomere length only in veterans exposed to severe combat stress [161]. In another study of former prisoners of war, Stein et al. [162] proposed that there might be a unique contribution of different specific types of traumatic stress across the lifespan on telomere length in later life. However, other studies supporting an association between PTSD and shorter telomere length did not find an effect of trauma type on this association [163]. Similarly, history of traumatic brain injury (TBI) may play a major confounding role on oxidative equilibrium of the brain as it is regularly associated with oxidative damage and has been, therefore, implicated in PTSD development, representing a robust PTSD risk factor [164,165].

Furthermore, several studies also reported negative findings and failed to show differences between PTSD and non-PTSD individuals exposed to traumatic stress. For example, another Croatian research group failed to show differences in urinary 8-OH-DG, serum Thromboxane B2, and urates between veterans with vs. without PTSD [166], discussing potential confounding effects of medication and comorbidity (e.g., depression). Interestingly, recent studies showed even converging results toward an up-regulation of antioxidants levels/function. For example, Michels et al. [167] found increased GSH levels by 23% in PTSD cases compared to HC, while later on, Ogłodek reported increased plasma levels of GSH-Rd and HO-1, but reduced PON-1 levels in PTSD patients vs. HC, with the most pronounced aberrations in PTSD individuals with comorbid severe depression [168,169].

The above mentioned, contradictory at first sight findings, are indicative of the complexity characterizing the oxidative mechanisms and the consequent difficulty in their comprehension. Specifically, this complexity appears to be related to several factors such as the variation of the nature of the traumatic incident, the interim between the traumatic incident and the study, the fulfillment or not of the diagnostic criteria of a psychiatric disorder, the comorbidity, the age of the subjects when exposed to trauma, the particular role of each antioxidant in the oxidative cascade, and the evaluation of circulating levels vs. activity of the antioxidant, just to name a few. Thus, someone needs to be cautious before extrapolating definitive assumptions.

**Table 3:** Summary of clinical findings on traumatic-stress-related OXS measures by exposure type.

Redox Index	Outcome	References	ELS/PTSD
**ROS/RNS**	NO ↑	[143]	PTSD
	ONOO^−^ ↑	[143]	PTSD
**Antioxidants levels**	GSH ↑	[167]	PTSD
**Antioxidant activity**	GSH-Px ↓	[132] [144]	ELS PTSD
	GSH-Px ↑	[135] [147]	ELS PTSD
	GSH-Rd ↑	[168,169]	PTSD
	SOD ↑	[132]	ELS
	SOD Ø	[147]	PTSD
	SOD ↓	[144]	PTSD
	PON-1 ↓	[146,168,169]	PTSD
	HO-1 ↑	[168,169]	PTSD
	TRAP ↓	[132]	ELS
**Redox end-products**	Carbonyl ↑	[132]	ELS
	MDA ↑	[146]	PTSD
	8-OH-DG ↑	[133]	ELS
	8-OH-DG Ø	[166]	PTSD
	OxLDL ↑	[169]	PTSD
	Iso-Ps ↑	[134]	ELS
	Thromboxane B2 Ø	[166]	PTSD
**Other redox-related parameters**	GLU ↑	[79]	PTSD
	GLU ↓	[149]	PTSD
	GLU Ø	[167]	PTSD
	NAA ↓	[77,79]	PTSD
	NAA Ø	[167]	PTSD
	GABA ↑	[167]	PTSD

8-OH-DG: 8-hydroxy-deoxy-guanosine; AMPK: neuronal 5′-adenosine monophosphate-activated protein kinase; CAT: catalase; COX-2: cyclooxygenase-2; ELS: early-life stress; GABA: γ-amino-butyric-acid; GLU: glutamate; GSH: glutathione; GSH-Px: GSH peroxidase; GSH-Rd: GSH reductase; HO-1: heme oxygenase-1; IsoPs: isoprostanes; MDA: malondialdehyde; NAA: N-acetyl-aspartate; NO: nitric oxide; ONOO^−^: peroxynitrite; OxLDL: oxidized low-density lipoprotein; PON-1: paraoxonase-1; PTSD: posttraumatic stress disorder; RNS: reactive nitrogen species; ROS: reactive oxygen species; SOD: superoxide dismutase; TRAP: total reactive antioxidant potential; ↑: induction; ↓: inhibition; Ø: no change.

## 4. Additional Neurobiological Pathways of Trauma-Related OXS

Results from the above-mentioned literature on basic and clinical research suggest that the neurobiological mechanisms involved in the interaction between traumatic and oxidative stress are highly complex. Indeed, in addition to OXS-related mechanisms described in Section 2, there are even more potential pathophysiological pathways particularly associated with traumatic-stress-related OXS.

### 4.1. Inflammation-Induced OXS

Inflammation is heavily involved in oxidative pathways and a vital mechanism of OXS generation (Figure 2), in particular in stress-related disorders [170], which are heavily associated with immune dysregulation [171]. Indeed, both animal and human studies provide evidence for an ELS-associated pro-inflammatory state later in life [172,173,174,175,176], while numerous studies published during the last decade repeatedly confirm, among others, elevated levels of circulating pro-inflammatory cytokines [e.g., tumor necrosis factor (TNF)-α; interferon (IFN)-γ], white blood cells, and CRP in individuals with PTSD across various trauma types [177,178,179].

Inflammatory signaling (e.g., through cytokines) stimulates the synthesis of NO via NOS isoforms (eNOS from endothelial cells, iNOS from peripheral lymphocytes, and nNOS from neuronal tissue), the NOX-mediated oxidative cascade, and nuclear factor-kappa beta (NF-kB) activation [180,181]. Thereby, among the different NOS isoforms, iNOS is of particular interest since it is induced by the pro-inflammatory cytokines IFN-γ and TNF-α secreted in response to local inflammation [181,182]. Interestingly, local inflammation is initiated by the activation of toll-like receptors (TLR) by pathogen-associated molecular patterns (PAMPs), which, in turn, subsequently activate the NF-kB as well as subsequent cascades, finally leading to the secretion of not only pro-inflammatory cytokines, chemokines and prostaglandins, but also ROS/RNS [183,184].

In addition, inflammation influences the redox equilibrium also through the kynurenine pathway [185]. Thereby, pro-inflammatory signaling triggered by chronic or intensive stress can activate the kynurenine 3-monooxygenase (KMO) enzyme, which directs the tryptophan metabolic pathway towards quinolinic acid formation [186]. The latter constitutes a potent generator of GLU, resulting in NMDA-R overactivation and excitotoxicity as well as iNOS induction and intracellular NAD decrease, culminating in RNS generation and lipid peroxidation [187]. For a detailed review of the tightly synergistic interaction of the redox dysregulation, (neuro-) inflammation, and NMDA-R function in the context of psychiatric disorders, please refer to Steullet et al. [188].

### 4.2. Glucocorticoid-Induced OXS

Disorders associated with traumatic stress exert a major and chronically dysregulating effect on the (re)activity of the hypothalamic-pituitary-adrenal (HPA) axis [4,5,189,190]. However, glucocorticoids (GCs), as the end-effector molecules of the HPA axis, have a vital modulating effect on inflammation and OXS production. Typically, GCs are known to exert an inhibitory effect on both the inflammatory molecules and the NOX-derived ROS production [191,192]. In parallel, GCs upregulate antioxidant defenses through genomic and non-genomic mechanisms [193]. However, under conditions of chronic/repeated stress, persistently dysregulated GCs modulation has been associated with pro-inflammatory cytokine production [194,195]. This immune regulatory aspect of GCs effects along with their role in GLU induction and Ca upregulation can equivocally increase mitochondrial respiration and oxidative phosphorylation, potentially escalating to OxS [196,197]. For example, chronic subcutaneous corticosterone administration was associated with increased OXS and reduced antioxidant enzyme activity in the rat hippocampus [198], while a meta-analysis of 19 studies suggested a positive correlation between duration of GCs exposure and OXS brain damage in vertebrates [199]. Interestingly, a recent human gene expression study in Veterans with PTSD has also supported an indirect interplay between HPA axis and OXS by showing significant associations and differential expressions of both the GC-receptor (GR) gene NR3C1, and the TXN-Rd1 gene with PTSD [200].

### 4.3. Epigenetic Mechanisms

Recent literature has implicated acute stress-related epigenetic mechanisms in the generation of OXS. Proposed epigenetic mechanisms in a preclinical level relate to the alteration of acetylation of the BDNF gene by histones H3 and H4 in the hippocampus and amygdala [201], direct modification of DNA methylation status [202], methylation of the GR gene NR3C1 [203,204], and acetylation on the Lysine residue 27 of the metabotropic GLU-R2 [205]. On the other hand, clinical studies on holocaust survivors provided evidence of methylation of the FKBP-5 gene-regulator of GR [206] and methylation of the NR3C1-1F promoter region of the GR gene in PTSD patients [207] with even transgenerational impact [208,209]. Finally, a recent study among Iraq and Afghanistan Veterans showed that PTSD symptoms, as well as pain severity and sleep disturbance, were all significantly associated with the DNA methylation age regulated by a defective Klotho gene SNP variant rs9315202 [210]. BDNF, GR, FKBP-5, GLU-R, and Klotho genes are all suggested as effector components of the oxidative system, and epigenetic changes in those genes may additionally mediate the trauma-related repercussions on the redox equilibrium.

### 4.4. Circadian Dysregulation and Melatonin

Circadian dysregulation and sleep disturbances represent a core feature of traumatic-stress-related disorders, often closely related to the severity of PTSD psychopathology [211,212,213]. Circadian dysregulation after traumatic stress can lead to a breakdown of biological adaptive mechanisms and may constitute another crucial link between traumatic stress and OXS [214,215]. Normally, a proper temporal order of biological systems is associated with balanced restorative properties throughout the body, allowing antioxidant processes to take over [216,217]. On the other hand, both preclinical [218,219] and clinical studies [220] show increased OXS marker levels, consequent to laboratory-induced sleep deprivation and circadian dysregulation, respectively [221,222].

Besides potentially disrupted circadian properties of the HPA-axis and GC-signaling implicated in the redox equilibrium machinery, there is a large amount of data suggesting that the pineal effector of the central circadian system and essential synchronizing hormone melatonin (MLT) also exhibit distinct antioxidant characteristics [223,224]. Given its small molecular size and its amphipathic behavior, MLT efficiently protects every subcellular compartment against OXS through a variety of mechanisms [225]. MLT and its metabolites exert direct scavenging effects on ROS/RNS and radical products; reduce free radical formation by support of mitochondrial electron flux, induce redox, and other antioxidant enzymes while suppressing pro-oxidant enzymes; contribute to the maintenance of membrane stability; protect mtDNA and homeostasis; reduce metal-induced toxicity through concurrent chelating cascades; and finally induce defense mechanisms suppressing inflammation [217,224,225,226,227,228,229,230]. MLT, thus, exerts substantial protective effects and is responsible for nocturnal tissue recovery after the daily free radical brain damage due to high oxygen utilization [231,232].

Disrupted MLT levels in the first 48 h after traumatic stress exposure were shown to be associated with a higher PTSD development risk [233]. This is particularly interesting as several animal models have shown that melatonin can effectively counteract OXS and neurodegeneration, suppress various apoptotic markers, and ameliorate the oxidant effects of GCs induced by stressful conditions [234,235,236,237]. MLT has been especially shown to protect hippocampal neurons from oxidative stress, by preventing GC-related toxicity through a decrease of receptor translocation to nuclei in models of both sleep deprivation and chronic stress [236,238,239,240].

## 5. PTSD Comorbidities as OXS Moderators

ELS/CT history, as well as PTSD, have all been associated with a large number of mental and physical comorbidities.

### 5.1. Depression

One of the highest comorbidities seen after ELS/CT and in PTSD is major depression [241]. Both disorders share common pathophysiological pathways [242,243], while depression has also been repeatedly associated with oxidative dysregulation in CNS and the periphery [170,244], possibly suggesting an additive effect on OXS in this comorbidity [245,246]. This presumption is furthered by two case studies conducted by the same research group on subjects diagnosed with depression alone of various severity levels, PTSD alone, as well as depression plus PTSD [168,169]. The measured inflammatory (IL-18, IL-33, MIP) and oxidative parameters (PON-1, GSH-Rd, OxLDL, iNOS, HO-1) in all patient groups deviated from HC, with the most deviation being evidenced by the PTSD group with comorbid depression.

### 5.2. Traumatic Brain Injury (TBI)

PTSD also shows a high comorbidity rate with TBI [247]. Both PTSD and TBI commonly occur in the general population but are especially comorbid in military populations [248]. TBI shares overlapping pathophysiological pathways with PTSD (i.e., altered brain networks with reduced prefrontal function, volume loss in amygdala, etc.) [249,250,251], leading to similar symptoms (e.g., cognitive impairment, sleep disruption), higher levels of PTSD symptoms, and increased vulnerability to the disorder [248,252,253,254,255]. Prospective studies have confirmed TBI as a major predictor of PTSD risk [256]. The pathogenesis of TBI is directly related to secondary biochemical cascades following injury and exacerbating primary damage, which result in the imbalance between oxidant agents and antioxidant defense and, thus, lead to OXS, excitotoxicity, ionic imbalances, cerebral edema, neuroinflammation, neural dysfunction, and cell loss with functional impairment [257,258,259]. Interestingly, TBI in rats has been found to cause increased DNA methylation and reduced expression of the *Aanat* gene [260], encoding serotonin N-acetyltransferase, one of the two enzymes involved in the synthesis of melatonin from serotonin.

### 5.3. Other Comorbidities

PTSD has also been frequently related to several further co-morbidities, such as chronic fatigue syndrome (CFS) [261,262,263], fibromyalgia [264,265,266,267,268], irritable bowel syndrome (IBS) [269], and rheumatoid arthritis [270], which all share a very similar underlying neuroendocrinological profile to PTSD (e.g., hypocortisolism, blunted diurnal GC rhythm and HPA axis reactivity) [271,272,273,274,275] and have all been repeatedly associated with increased OXS [276,277,278,279,280,281,282].

## 6. Discussion

Traumatic stress may chronically affect master homeostatic systems at the crossroads of peripheral and central susceptibility pathways (e.g., HPA axis, autonomic nervous system, circadian system, metabolic system, and immune system) and lead to the biological embedment of trauma-related allostatic trajectories through neurobiological alterations (i.e., gene expression, glucocorticoid signaling, epigenetic changes) even decades later. The bulk of evidence suggests an imbalance of pro-/anti-oxidative mechanisms under conditions of traumatic stress, respectively leading to a systemic oxidative dysregulation accompanied by toxic oxidation byproducts. Higher OXS may be a major allostatic trajectory linking exposure to traumatic stress with mental and somatic comorbidity, as both ELS/CT and PTSD have been repeatedly associated with several comorbid mental and somatic disorders, also associated with higher OXS levels. Yet, there is still a substantial heterogeneity in relative findings, probably due to several confounding parameters, as well as to the equivocal directionality of not only the involved oxidative mechanisms but other major homeostatic ones (e.g., inflammation, HPA-axis, circadian system).

First, the inherent difficulty in the translation of animal studies’ findings into human biology hampers the enlightenment on the trauma-related oxidative puzzle. In addition, different central (i.e., CNS) and peripheral mechanisms pose significant complications in the generalization of the outcomes. This may also be a fact within the CNS, as antioxidants’ levels may vary depending on the brain topography, while oxidative biomarker levels do not always necessarily reflect the antioxidants’ activity. Another inherent difficulty in further elucidating the traumatic-stress-induced oxidative neurobiological aberrations stems from the activation of the same or similar oxidative and systemic pathways in several stress-related nosological entities (i.e., depression, anxiety, sleep disorders, and PTSD). This means that apart from the trauma itself, other psychiatric symptoms could separately activate oxidative mechanisms as well, while this dysregulation may also be based on shared genetic vulnerability [283].

Additional factors possibly explaining the heterogeneity of the trauma-related OXS findings may relate to the differential type, intensity (i.e., including individual subjective appraisal), chronicity, frequency, time since exposure, and developmental timing of the traumatic stress exposure, possibly affecting consequent oxidative mechanisms in a differential way and resulting in different redox equilibrium states [4]. Also, sociodemographic, gender-specific, and lifestyle factor variation can confound the outcome with heterogeneity [284]. Having spoken about lifestyle, researchers should be aware that patients with traumatic stress-related disorders may be more likely to engage in unhealthy diet and behaviors, such as smoking, alcohol consumption, and sedentary lifestyle [285,286], all of them closely linked to pro-inflammatory states and higher OXS [45,287,288,289,290].

In addition, the cross-sectional design regularly used in related studies represents another crucial limitation factor hampering the elucidation of the causality direction between traumatic stress exposure and OXS. It is, indeed, unclear whether higher OXS levels in victimized children or adults reflect the effects of traumatic stress exposure per se, rather than a pre-existing (genetic or functional) risk factor similar to other psychiatric disorders [291,292]. Finally, differences in trauma and symptom assessment methods may play a crucial role in explaining the heterogeneity of the trauma-related OXS findings.

## 7. Conclusive Remarks

The neurobiological mechanisms, underpinning the interaction between traumatic stress and OXS, are highly complex. The exact interplay among oxidative parameters and other systemic components in the context of trauma needs to be better elucidated. We encourage researchers to take into consideration the many confounding parameters reviewed analytically in this review in order to increase their comprehension of the neurobiological substrates linking traumatic stress, OXS, and disease, and potentially enhance new OXS-related therapeutic or even preventive strategies for exposed individuals.

## Figures and Tables

**Table 1:** Definitions of trauma-related terminology.

Term	Definition
**Traumatic Event**	An event that threatens actual or perceived injury, death, or the physical integrity of self or others and also causes horror, terror, or helplessness at the time it occurs and overwhelms a person’s ability to cope (e.g., physical/sexual abuse, medical trauma, motor vehicle accident, acts of terrorism, war experiences, natural and human-made disasters, witnessed homicides/suicides) [10].
**Early Life Stress (ELS)**	A broad spectrum of adverse and stressful experiences (e.g., maltreatment, neglect, parental separation, parental loss, extreme poverty, starvation, domestic/community/school violence, medical trauma/illness, war and disaster experiences, etc.) during the first months of life, early and late childhood, and adolescence [11], while the term has been recently extended by some authors and includes also prenatal life events [12].
**Childhood Trauma/** **Maltreatment (CT)**	A more specific form of ELS restrictively referring to only physically or emotionally painful or distressful interpersonal traumatic events during childhood (e.g., physical/sexual/emotional abuse, physical/emotional neglect) [13].
**Childhood Adverse** **Experiences (ACEs)**	This broader term includes both ELS and CT. All ACEs exhibit a dose-response effect between number and duration of ACEs and related negative health effects [14].
**Severe Life Stress (SLS)**	A serious psychosocial event of random duration, with the potential of causing an impacting psychological traumatism and producing severe strain (e.g., loss of loved ones, job loss, prolonged social isolation, etc.) [15].
**Posttraumatic Stress** **Disorder (PTSD)**	A trauma- and stress-related disorder with distinctive symptoms following a psychologically distressing event outside the range of usual human experience [10]. Diagnostic criteria include current symptoms from each of four symptom clusters: intrusion, avoidance, negative alterations in cognitions and mood, and alterations in arousal and reactivity including sleep disturbances. The estimated lifetime prevalence of PTSD in the general U.S. population lies between 5–6% in men and 10–14% in women [16]. The previously defined as Secondary Traumatic Stress (STS) condition is now considered a valid DSM-5 Criterion A for PTSD.

**Table 2:** Summary of preclinical findings on traumatic-stress-related OXS measures by animal model.

Redox Index	Outcome	References	Stress Model
**ROS/RNS**	ROS ↑	[105,107] [117] [125]	MS PPE Combined model
	O_2_^●−^ ↑	[105] [117]	MS PPE
	H_2_O_2_ ↑	[112]	SI
	NO ↑	[116]	PPE
	ONOO^−^ ↑	[117]	PPE
	NOX ↑	[106] [116] [119] [122] [123]	MS PPE SPS IFS RSE
	NOS ↑	[115,116]	PPE
	iNOS ↑	[121] [123]	SPS RSE
	Mitochondrial activity ↑	[103]	MS
**Antioxidants levels**	GSH ↑	[105]	MS
	GSH ↓	[119]	SPS
	NO ↑	[118]	SPS
**Antioxidant activity**	CAT ↓	[104,108] [112] [113]	MS SI MCD
	CAT ↑	[129] [130]	MS SI
	TAC ↑	[108] [112]	MS SI
	SOD ↑	[129] [130]	MS SI
	SOD ↓	[112] [113] [119]	SI MCD SPS
	GSH-Px ↓	[107,108] [112,131] [119]	MS SI SPS
	GSH-Px ↑	[129] [114]	MS Prenatal stress
	GSH-Rd ↓	[131]	SI
**Redox end-products**	TBARS ↑	[104,129]	MS
	Carbonyl ↓	[113]	MCD
	Lipid peroxide ↑	[115]	PPE
	MDA ↑	[119]	SPS
	8-OH-DG ↑	[122]	IFS
**Other redox-related parameters**	PVI activity ↓	[109] [110] [111]	PMS MS SI
	pAMPK ↑	[120]	SPS
	GABA progenitors ↓	[109]	PMS
	GLU ↓	[112]	SI
	NAA ↓	[112]	SI
	p-AMPK ↑	[122]	IFS
	COX-2 ↑	[118]	SPS
	PGE-2 ↑	[118]	SPS
	GAD-67 ↓	[122]	IFS
	p38 ↑	[121]	SPS

8-OH-DG: 8-hydroxy-deoxy-guanosine; AMPK: neuronal 5′-adenosine monophosphate-activated protein kinase; CAT: catalase; COX-2: cyclooxygenase-2; GABA: γ-amino-butyric-acid; GAD-67: glutamic acid decarboxylase-67; GLU: glutamate; GSH: glutathione; GSH-Px: GSH peroxidase; GSH-Rd: GSH reductase; H_2_O_2_: hydrogen peroxide; IFS: inescapable foot shocks; MCD: maternal care deprivation; MDA: malondialdehyde; MS: maternal separation; NAA: N-acetyl-aspartate; NO: nitric oxide; NOS: nitric oxide synthase; iNOS: inductible NOS isoform; NOX: nicotinamide adenine dinucleotide phosphate (NADPH) oxidase; O_2_^●−^: superoxide anion; ONOO^−^: peroxynitrite; p-38: protein-38 mitogen-activated protein kinase; PGE-2: prostaglandin E-2; PMS: prenatal maternal stress; PPE: prolonged predator exposure; PVI: GABAergic parvalbumin interneurons; RNS: reactive nitrogen species; ROS: reactive oxygen species; RSE: repeated stress exposure; SI: social isolation; SOD: superoxide dismutase; SPS: single prolonged stress; TAC: total antioxidant capacity; TBARS: thiobarbituric reactive substances; ↑: induction; ↓: inhibition.

## Data Availability

Not applicable.
